# Impaired insight in schizophrenia: impact on patient-reported and physician-reported outcome measures in a randomized controlled trial

**DOI:** 10.1186/s12888-022-04190-w

**Published:** 2022-08-28

**Authors:** Paul H. Lysaker, Peter J. Weiden, Xiaowu Sun, Amy K. O’Sullivan, Joseph P. McEvoy

**Affiliations:** 1 Richard L. Roudebush VA Medical Center and Indiana University School of Medicine, 1481 West 10th Street, Indianapolis, IN 46202 USA; 2 Alkermes, Inc., Waltham, MA USA; 3 Psychiatry and Health Behavior at Augusta University, Augusta, GA USA

**Keywords:** Insight, PANSS, Patient-reported outcome, Neurocognition, Quality of life, Schizophrenia, SF-12, CATIE schizophrenia trial

## Abstract

**Background:**

Impaired insight poses a challenge in the treatment of patients with schizophrenia because of its potential to jeopardize therapeutic engagement and medication adherence. This study explored how insight impairment, graded from none to extreme, is related to patient-reported mental health status, depression, and neurocognition in schizophrenia.

**Methods:**

In a post hoc analysis of the Clinical Antipsychotic Trials of Intervention Effectiveness (CATIE) study (NCT00014001), insight was measured using the Positive and Negative Syndrome Scale (PANSS) Item G12 (lack of insight). Additional assessments for this analysis included the 12-Item Short-Form Health Survey (SF-12) Mental Component Summary (MCS), physician- and patient-reported Clinical Global Impression–Severity (CGI-S), MATRICS Consensus Cognitive Battery, and Calgary Depression Scale for Schizophrenia. Relationships between patient-reported outcomes and PANSS total and Item G12 ratings were evaluated.

**Results:**

Among 1431 CATIE study participants in this analysis, increasingly impaired insight at baseline was significantly associated with better patient-reported quality of life (QoL), lower baseline depression, and greater divergence between physician- and patient-reported illness severity. Patients with more severely impaired insight reported milder illness compared with physician reports, particularly those with moderate-severe to extreme impairment (PANSS Item G12 rating ≥ 5), approximately 10% (138/1431) of CATIE participants. For the 90% of patients with PANSS Item G12 ratings < 5, patient-reported QoL decreased with increasing symptoms. SF-12 MCS scores were linearly related to baseline PANSS total score only in patients with PANSS total score < 90 (moderately ill or better), and better symptom scores were associated with higher QoL. No significant relationship between insight and neurocognition was observed.

**Conclusions:**

In the small subgroup (10%) of CATIE study patients with schizophrenia and PANSS Item G12 ratings ≥5, moderate-severe–severe/extreme insight impairment was associated with significantly more positive perception of QoL and illness severity by the patient versus the treating physician. This was not observed in the remaining 90% of patients with normal to moderately impaired insight, suggesting that poor insight as a threat to the validity of self-report is uncommon.

## Introduction

Between 50 and 80% of patients with schizophrenia are reported to have impaired insight into their illness [1–3]. In the context of a life-altering disease such as schizophrenia, retaining insight helps patients make sense of the meaning of life events. In contrast, poor insight could be understood as a failure to construct a coherent account of complex and potentially traumatic life experiences that can result in loss of functioning and hospitalization [4]. Insight is a multidimensional concept that includes awareness of illness, the capacity to re-label psychotic experiences as abnormal, and adherence to treatment, which vary along a continuum [5, 6]. Poor insight is a feature of schizophrenia across different cultures and across all stages of the illness, and it persists even after symptoms have remitted [2, 4]. From a treating clinician’s perspective, impaired insight is one of the most vexing aspects of the illness because of the challenges it poses for therapeutic engagement and medication adherence [7, 8].

The relationship between insight and health is complex. Poor clinical insight in schizophrenia has been associated with poorer medication adherence, which can lead to an increase in positive symptoms and relapse risk [2, 4, 9–12]. Conversely, poor insight also may exert a protective effect. In patients with schizophrenia or other psychotic illnesses, small but positive associations have been reported between insight and depression or suicidal thoughts or actions [9, 13–15]. However, more complex relationships between insight and suicidal thoughts or actions, mediated by other symptoms and changing over the course of illness, have been observed in several analyses [16, 17]. This dual nature of insight in schizophrenia outcomes has been called the insight paradox [4, 14, 18, 19]. Because symptoms of depression affect health-related quality of life (QoL), there may be an interaction between poor insight and better health-related QoL arising from a lower level of depressive symptoms. Negative impacts of depression on health-related QoL in patients with good insight may prevent these individuals from attaining personal goals and increase the risk of suicide [4, 19]. However, such determinations may be limited if insight is viewed by category (i.e., good versus poor insight) rather than as a continuum or assessed using only population means, which is typically how such data have been reported [20–22].

Neurocognition may also play a role in potential interactions between insight and mental health status, depression, and health-related QoL, although the specific connections between these domains and global insight are unclear. For example, one study in patients with psychotic disorders found that improvement in insight over 3 years was significantly associated both with fewer clinical symptoms and with better neurocognitive performance at baseline. Gradual improvement in insight over 3 years was associated with symptom improvement but not with improved neurocognition [23]. Additionally, a meta-analysis identified positive correlations between neurocognition and objective health-related QoL (i.e., observable life conditions) but negative or no association between neurocognition and subjective health-related QoL (e.g., patient-reported satisfaction with life conditions) [24].

To date, one of the major limitations of insight research is that insight generally has been conceptualized and reported as a categorical variable, whereas it seems more appropriate to consider insight as a dimensional phenomenon, lying along a continuum with gradations in the severity of lack of awareness [4]. As a result, it remains unknown whether there is a particular level of impaired insight (e.g., mild versus moderate versus severe) at which a lack of agreement with others’ appraisal of the patient’s well-being, neurocognitive abilities, and depression becomes apparent. The current post hoc analysis used data from the Clinical Antipsychotic Trials of Intervention Effectiveness (CATIE) [25] study to explore how insight, assessed both as a categorical variable (more versus less impairment) and based on gradations along a continuum from none to extreme, is related to patient-reported mental health status, depression, and neurocognition in patients with schizophrenia.

## Methods

We conducted a post hoc analysis using baseline, 6-month, and 12-month data from phase 1/1A of the National Institutes of Health (NIH)-supported CATIE study (NCT00014001) [25]. CATIE was conducted from January 2001 to December 2004 at 57 centers in the United States.

### Data source

The CATIE schizophrenia trial was a multiphase, randomized, controlled trial that compared the effectiveness of first- and second-generation antipsychotic medications in patients with schizophrenia for up to 18 months of treatment [25, 26]. Results from this landmark study are reported in numerous publications; the study design is therefore described here briefly. In phase 1 of CATIE, patients were randomly assigned to receive olanzapine, perphenazine, quetiapine, risperidone, or ziprasidone under double-blind conditions and were followed for up to 18 months or until discontinuation of assigned medication, whichever came first. Patients with tardive dyskinesia at study entry were excluded from the perphenazine arm and were randomly assigned to olanzapine, quetiapine, risperidone, or ziprasidone (phase 1A). Data from phase 1/1A were pooled. Further details on the rationale, design, and study methods of CATIE have been described previously [25, 26].

### Outcome measures

Baseline patient demographics and socioeconomic characteristics from the pooled phase 1 and phase 1A CATIE data included antipsychotic medication, age, sex, race, marital status, patient education, employment status, and living arrangements. Baseline insight was assessed using the Positive and Negative Syndrome Scale (PANSS) Item G12 (lack of judgment and insight). This single-item scale is a commonly used measure in published studies of insight [27] and is reported to be sufficiently sensitive to distinguish patient-rated functioning and symptom severity in schizophrenia [22, 28]. Significant correlations have been established between PANSS Item G12 and multidimensional insight scales, including the clinician-rated VAGUS Insight Into Psychosis scale [29], the Schedule for the Assessment of Insight–Expanded [30], and the Insight and Treatment Attitudes Questionnaire [31]. PANSS Item G12 is rated from 1 to 7 (1 = absent, 2 = minimal, 3 = mild, 4 = moderate, 5 = moderate-severe, 6 = severe, and 7 = extreme) [32], where higher scores indicate increasing symptom severity. In scoring PANSS Item G12, clinicians consider several dimensions of clinical insight, including awareness or understanding of the disorder, acknowledgment of common symptoms of schizophrenia, and awareness of the need for psychiatric treatment [22]. For this analysis, the goal was to determine whether there was a level of impairment along the Item G12 rating continuum that presented noticeable and clinically relevant insight challenges.

The primary outcomes of interest were patient-reported evaluation of mental and physical health (at baseline and at 6 and 12 months), as measured by the 12-Item Short-Form Health Survey (SF-12) Mental and Physical Component Summary (MCS and PCS), where lower scores indicate poorer health-related QoL [33], and a patient-reported overall mental/emotional health status score at baseline from a single-item, stand-alone query: “Please rate your current state of mental or emotional health by choosing a number from 1 to 100,” where a score of 1 is the worst possible state and a score of 100 is the best possible state. The association between baseline insight level and patient-reported schizophrenia symptom severity at baseline was also examined by comparing patient-reported severity of illness (assessed using the Patient Global Impression−Severity [PGI-S] scale) [34] versus physician-reported severity of illness (assessed using the Clinical Global Impression−Severity [CGI-S] scale). To better characterize the clinical impact of different levels of insight, we also examined the relationship between baseline scores on neurocognition, depression, and schizophrenia symptom scales and baseline PANSS Item G12 ratings in the CATIE population. Those assessments included PANSS components identified through an analysis of patients at the West Haven Veterans Affairs Medical Center (i.e., negative, positive, cognitive, emotional discomfort, and hostility) [35], neurocognition measured by the Neurocognitive Composite Score (MATRICS Consensus Cognitive Battery [MCCB]) [36], and scores of depression measured by the Calgary Depression Scale for Schizophrenia [37].

### Statistical analyses

Patient characteristics and demographics were summarized using means and SDs for continuous variables and frequency and percentages for categorical variables. Associations between baseline PANSS Item G12 rating and baseline scores on patient-reported outcomes, symptoms scales, and neurocognitive scales were explored using analysis of variance. Based on results of those analyses, patient characteristics and demographics were then compared for patients with PANSS Item G12 ratings of 1 to 4 (good or fair insight) versus those with ratings of 5 to 7 (poor insight) using *t* tests for continuous variables and Chi-square tests for categorical values.

Local regression (LOESS), a nonparametric technique that uses local weighted regression to fit a smooth curve through points of a scatterplot [38], was used to explore the relationship between SF-12 MCS and PANSS total scores using baseline data. To simplify the curve, a piecewise linear model was fit and the impact of poor insight was tested. A mixed model was used to evaluate and quantify the relationship between patient-reported change in SF-12 MCS score and PANSS Item G12 insight level change, as well as dichotomized PANSS Item G12 rating (< 5 versus ≥5), controlling for baseline SF-12 MCS score, baseline PANSS total score, and time point (6 versus 12 months). In all analyses, a 2-sided *P* < 0.05 was the threshold by which differences were statistically significant. SAS version 9.4 was used to conduct the analyses.

## Results

### Patient demographics and baseline scores

A total of 1431 patients with schizophrenia from the CATIE phase 1/1A study were included in this post hoc analysis; most patients were male (74.3%) and white (61.1%), and mean age was 40.6 years (Table 1). At baseline, mean (SD) SF-12 MCS score was 40.9 (11.7) and SF-12 PCS score was 48.2 (10.2).

**Table 1:** Baseline characteristics and demographics of CATIE study participants by PANSS Item G12 insight rating

Characteristic	All Patients ***N*** = 1431	PANSS Insight Item Rating 1 to 4 (Absent to Moderate) ***n*** = 1293 (90%)	PANSS Insight Item Rating 5 to 7 (Moderate-Severe to Extreme) ***n*** = 138 (10%)	***P*** value*
Treatment for phase 1/1A, n (%^a^)				0.7710
Olanzapine	330 (23.1)	304 (92.1)	26 (7.9)	
Perphenazine	256 (17.9)	228 (89.1)	28 (10.9)	
Quetiapine	330 (23.1)	298 (90.3)	32 (9.7)	
Risperidone	333 (23.3)	299 (89.8)	34 (10.2)	
Ziprasidone	182 (12.7)	164 (90.1)	18 (9.9)	
Age, y, mean (SD)	40.6 (11.1)	40.7 (11.0)	39.6 (12.1)	0.2815
Age group, n (%^a^)				0.0998
18–35 years	449 (31.4)	395 (88.0)	54 (12.0)	
36–45 years	476 (33.3)	438 (92.0)	38 (8.0)	
46+ years	506 (35.4)	460 (90.9)	46 (9.1)	
Sex, n (%^a^)				0.0820
Male	1063 (74.3)	952 (89.6)	111 (10.4)	
Female	368 (25.7)	341 (92.7)	27 (7.3)	
Race, n (%^a^)				0.2376
White	875 (61.1)	789 (90.2)	86 (9.8)	
Black	506 (35.4)	462 (91.3)	44 (8.7)	
Other	50 (3.5)	42 (84.0)	8 (16.0)	
Marital status, n (%^a^)				0.0478
Married	166 (11.6)	156 (94.0)	10 (6.0)	
Previously married	414 (28.9)	381 (92.0)	33 (8.0)	
Never married	851 (59.5)	756 (88.8)	95 (11.2)	
Patient education, n (%^a^)				0.2445
< 12 years	365 (25.5)	336 (92.1)	29 (7.9)	
12 years	506 (35.4)	449 (88.7)	57 (11.3)	
> 12 years	560 (39.1)	508 (90.7)	52 (9.3)	
Employed, n (%^a^)				0.3161
No	1213 (84.8)	1092 (90.0)	121 (10.0)	
Yes	218 (15.2)	201 (92.2)	17 (7.8)	
Lives alone, n (%^a^)				0.2536
No	1084 (75.8)	974 (89.9)	110 (10.1)	
Yes	347 (24.2)	319 (91.9)	28 (8.1)	

*Abbreviations*: *PANSS* Positive and Negative Syndrome Scale, *SD* standard deviation

*For comparisons between patients with PANSS Item G12 ratings of 1–4 versus 5–7, *t* tests were performed on continuous variables, and Chi-square tests were performed on categorical values

^a^For “All Patients,” percentages indicate proportion of total patients. For PANSS Insight subgroups, percentages indicate proportions of patients with the given characteristic

Table 2 presents baseline scores on health-related QoL, illness severity, depression, and neurocognitive scales by PANSS Item G12 ratings from the 1431 patients who had complete data for physician-rated measures and patient-reported outcomes at baseline. PANSS Item G12 ratings for lack of insight were 1 (absent) in 22% (*n* = 309), 2 (minimal) in 18% (*n* = 258), 3 (mild) in 27% (*n* = 387), 4 (moderate) in 24% (*n* = 339), 5 (moderate-severe) in 6% (*n* = 80), and 6 to 7 (severe to extreme) in 4% (*n* = 58) of patients (patients scoring 6 or 7 were grouped due to small sample size). Patients meeting the predefined criterion for impaired insight (PANSS Item G12 rating ≥ 5) at baseline represented 10% (*n* = 138) of the total population.

**Table 2:** CATIE population baseline scale scores by PANSS Item G12 insight rating

Assessments, mean (SD)	PANSS Item G12 Rating						
	1 Absent ***n*** = 309	2 Minimal ***n*** = 258	3 Mild ***n*** = 387	4 Moderate ***n*** = 339	5 Moderate-Severe ***n*** = 80	6/7 Severe/Extreme ***n*** = 58	***P*** value*
Patient-reported outcomes							
Mental/emotional health item	58.5 (26.3)	58.0 (26.1)	59.1 (26.1)	59.0 (27.7)	72.7 (26.7)	74.1 (28.5)	< 0.0001
SF-12 PCS	47.7 (10.8)	47.7 (10.1)	48.1 (10.6)	48.3 (9.9)	50.5 (7.8)	50.3 (8.1)	< 0.0001
SF-12 MCS	39.7 (12.0)	40.2 (11.0)	40.0 (11.5)	41.4 (11.2)	44.4 (11.9)	49.1 (11.7)	< 0.0001
Schizophrenia severity							
CGI-S	3.7 (1.0)	3.8 (0.9)	3.9 (0.9)	4.2 (0.9)	4.5 (1.0)	4.6 (1.0)	< 0.0001
PGI-S	3.5 (1.5)	3.6 (1.6)	3.6 (1.5)	3.6 (1.6)	3.0 (1.7)	2.6 (1.7)	< 0.0001
PANSS components^**^							
Negative	17.5 (6.4)	20.1 (5.8)	21.6 (6.1)	23.3 (6.9)	24.1 (7.2)	27.1 (8.0)	< 0.0001
Positive	14.2 (5.3)	15.5 (4.8)	17.0 (4.8)	18.7 (5.7)	19.1 (6.0)	20.0 (6.2)	< 0.0001
Cognitive	13.5 (4.2)	16.5 (4.2)	18.2 (4.0)	20.6 (4.4)	23.2 (4.7)	24.8 (5.6)	< 0.0001
Emotional discomfort	10.6 (4.2)	11.2 (3.6)	11.4 (3.6)	11.2 (3.9)	10.3 (3.6)	9.1 (3.2)	< 0.0001
Hostility	6.0 (2.5)	6.7 (2.5)	7.5 (2.8)	7.5 (3.1)	8.1 (3.2)	7.8 (3.8)	< 0.0001
Neurocognitive Composite Score (MCCB)^***^	51.3 (9.8)	51.3 (9.7)	49.8 (9.3)	48.7 (11.1)	46.8 (10.0)	49.2 (9.7)	0.1648
Calgary total score	5.0 (4.8)	4.9 (4.3)	4.8 (4.4)	4.3 (4.3)	2.9 (3.7)	2.1 (2.4)	< 0.0001

*Abbreviations*: *CGI-S* Clinical Global Impression–Severity, *MCCB* MATRICS Consensus Cognitive Battery, *MCS* Mental Component Summary, *PANSS* Positive and Negative Syndrome Scale, *PCS* Physical Component Summary, PGI-S, Patient Global Impression–Severity, *SF-12* 12-Item Short-Form Health Survey

*Overall difference

^**^Based on West Haven Veterans Administration sample [35]

^***^MCCB was standardized to a baseline cohort from 5 other component scores (mean = 0; standard deviation = 1). The T-score reported was calculated as 50 + 50 + 10 × Neuro_SS, a normative value used for MCCB

At baseline, scores for most outcomes varied significantly with level of insight (PANSS Item G12; *P* < 0.0001). In general, greater physician-rated symptom severity and impairment in functional outcomes were observed for patients with greater lack of insight at baseline (Table 2). Conversely, patients with greater insight impairment had lower levels of depression (Calgary Depression total score) compared with patients with lower levels of impairment. Neurocognition (based on the MCCB) did not appear to be significantly related to insight (Table 2).

### Insight impairment and physician-reported versus patient-reported schizophrenia severity

**Fig. 1:**
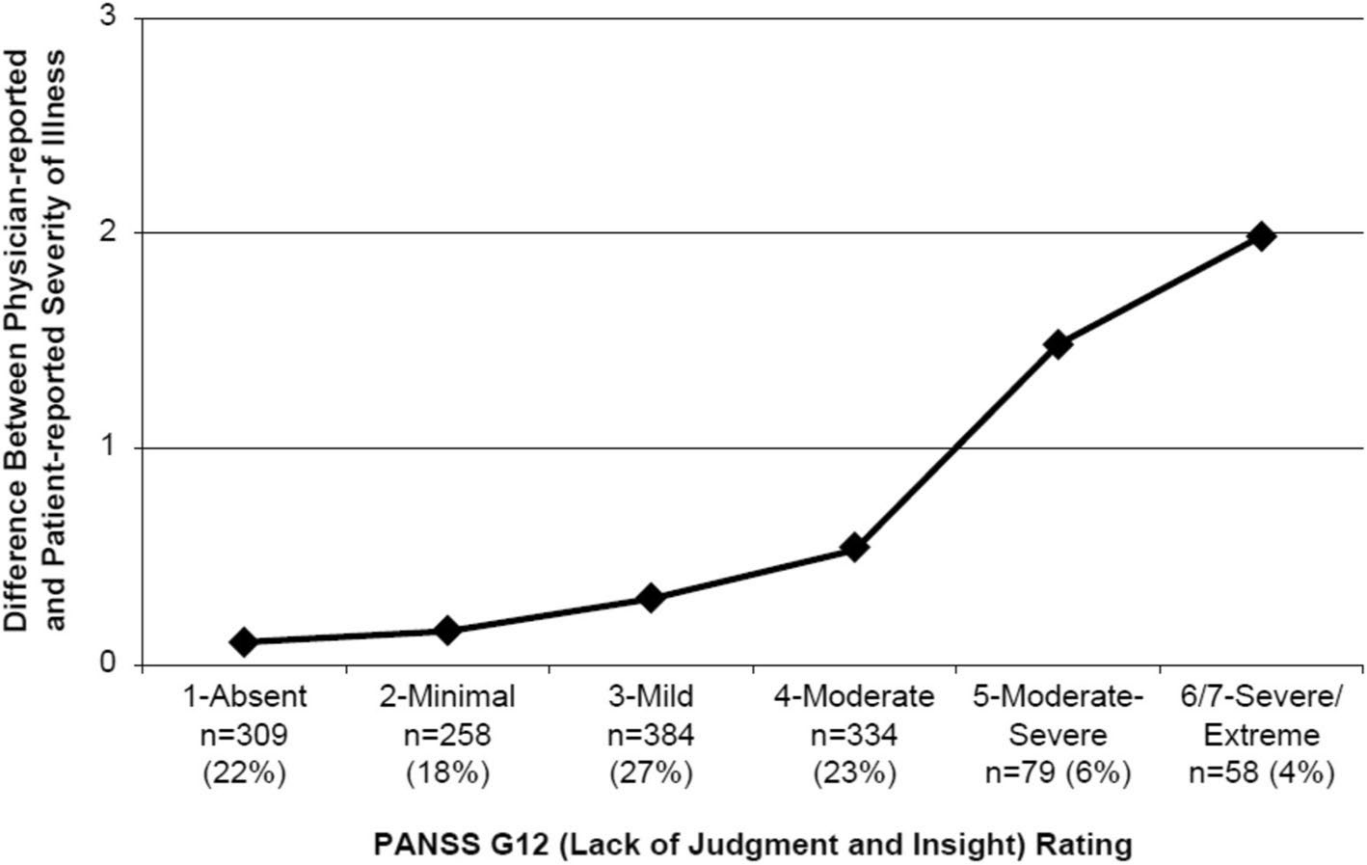
Insight impairment and perceived schizophrenia severity: difference^*^ between physician- and patient-reported severity of illness. Abbreviations: CGI-S, Clinical Global Impression–Severity; PANSS, Positive and Negative Syndrome Scale; PGI-S, Patient Global Impression–Severity. *Calculated as mean CGI-S score – mean PGI-S score

Level of insight at baseline was significantly associated with both physician- and patient-reported illness severity scores at baseline (both *P* < 0.0001; Table 2). However, whereas physician-reported schizophrenia severity (CGI-S) was higher for patients with the most severe levels of impaired insight, these patients had the lowest patient-reported illness severity (PGI-S) (Table 2). Consequently, the difference between physician- and patient-reported schizophrenia severity was greatest for patients with the greatest impairment in insight (Fig. 1 and Table 2). The divergence between physician and patient ratings appeared to become clinically relevant (1.5 to 2 points in magnitude) for patients with moderate-severe to extreme lack of insight (Item G12 rating ≥ 5; Fig. 1). For the remaining patients (Item G12 rating < 5), mean CGI-S and PGI-S scores were comparable, within approximately 0.5 points of each other at each level of impairment.

### Relationship between insight impairment and patient-reported outcomes

**Fig. 2:**
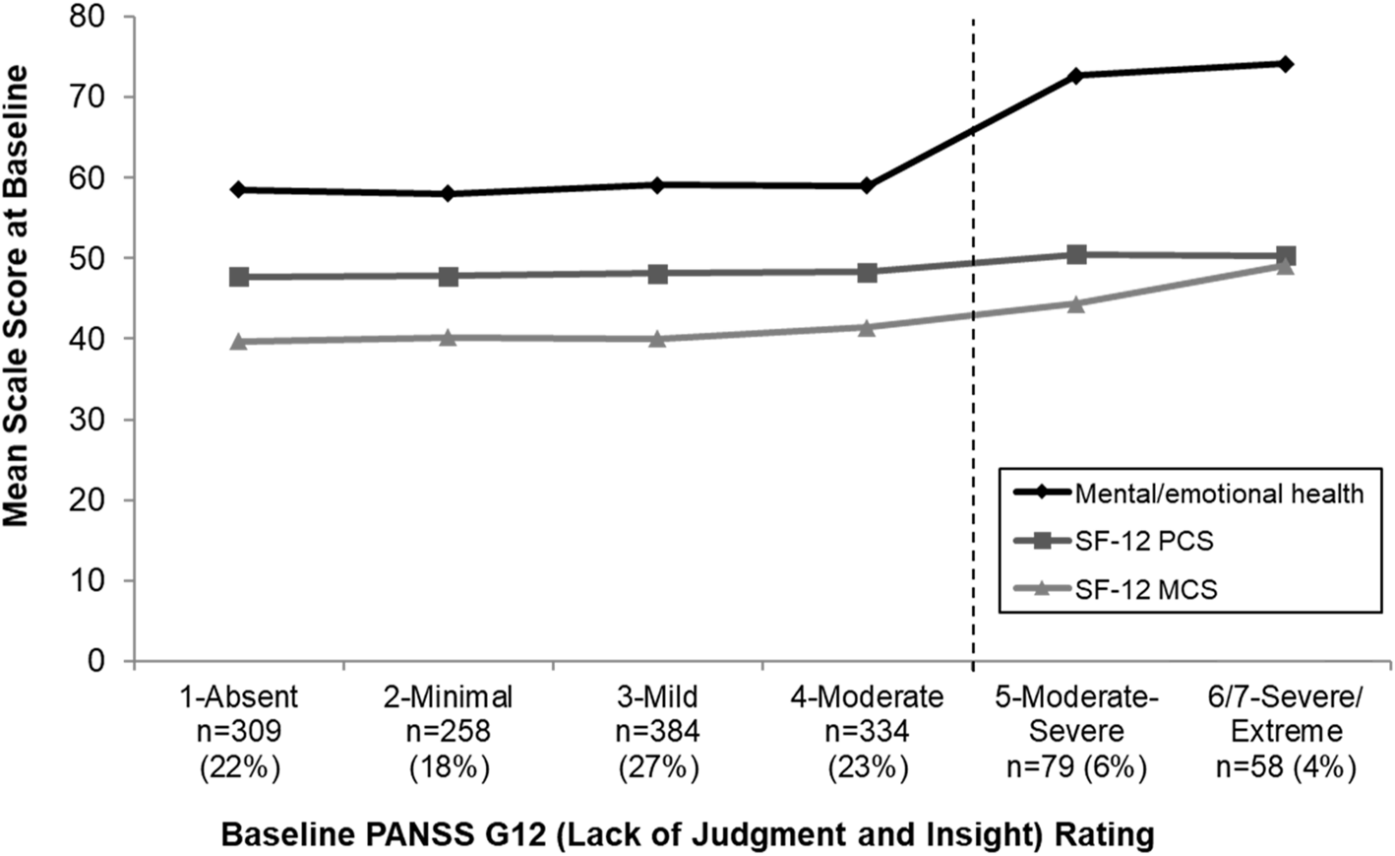
Patient-reported mental and physical health by insight score. Abbreviations: MCS, Mental Component Summary; PANSS, Positive and Negative Syndrome Scale; PCS, Physical Component Summary; SF-12, 12-Item Short-Form Health Survey

Baseline scores for all patient-reported outcomes varied significantly with level of insight (all *P* < 0.0001; Table 2). Specifically, patients with poorer insight had higher (better) scores on the mental/emotional health item, SF-12 MCS, and SF-12 PCS, with most apparent differences between patients with PANSS Item G12 ≥ 5 versus < 5 (Fig. 2 and Table 2).

Based on the observed difference between patients with moderate-severe to extreme insight impairment (PANSS Item G12 rating ≥ 5) and those with absent to moderate impairment on the mental/emotional health item, SF-12 MCS, and CGI-S and PGI-S scores, baseline demographic characteristics were explored by PANSS Item G12 ≥ 5 versus < 5. The two groups were comparable on all baseline characteristics except marital status (Table 1).

### Relationship between patient-reported SF-12 MCS and PCS scores and PANSS total score

**Fig. 3:**
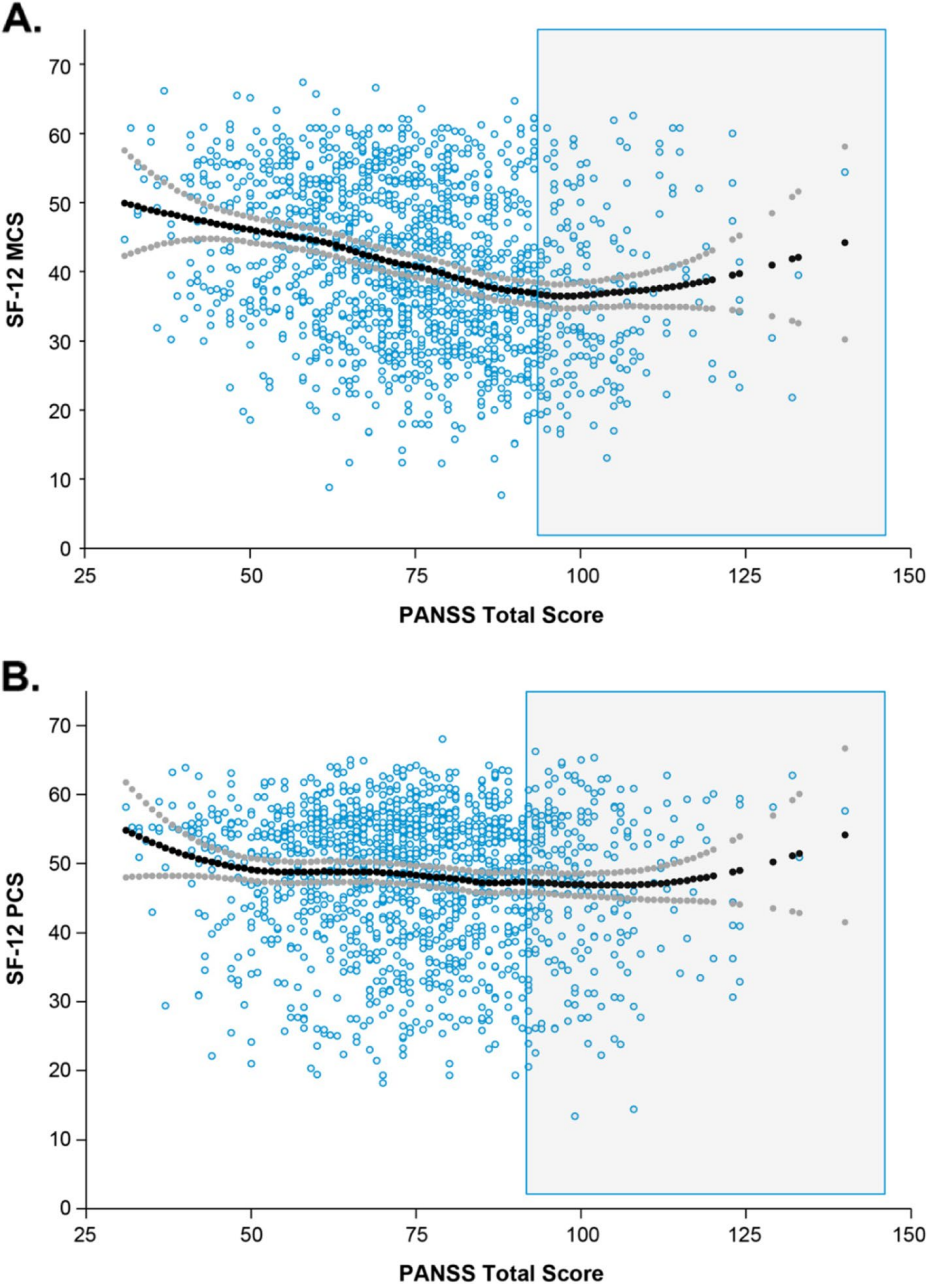
LOESS relationships between patient-reported outcomes and PANSS total score. Shown are LOESS relationships between patient-reported outcomes SF-12 MCS (A) and SF-12 PCS (B) and PANSS total score. Abbreviations: LOESS, locally weighted scatterplot smoothing; MCS, Mental Component Summary; PANSS, Positive and Negative Syndrome Scale; PCS, Physical Component Summary; SF-12, 12-Item Short-Form Health Survey

LOESS regression analysis suggested a piecewise linear relationship between SF-12 MCS score and PANSS total score, but no piecewise linear relationship between SF-12 PCS score and PANSS total score (Fig. 3); therefore, no further analyses were conducted using SF-12 PCS scores. The relationship between level of insight impairment and MCS score was assessed using a piecewise linear model. In the model with baseline PANSS data, when the baseline PANSS total score was <90, the SF-12 MCS score decreased by 2.6 points for each additional 10-point increase in the baseline PANSS total score. For example, a patient with a baseline PANSS total score of 80 would be expected to have an SF-12 MCS score 2.6 points lower than a patient with a baseline PANSS score of 70 (i.e., worse SF-12 MCS score was associated with more severe disease as measured by PANSS score). When the baseline PANSS score was ≥90, there was no significant relationship between PANSS total score and SF-12 MCS score.

In the model with longitudinal data, the SF-12 MCS score increased by 2.2 points (i.e., improved) for each 10-point decrease in PANSS total score from baseline to 6 months (Table 3). Therefore, if a patient’s PANSS score fell from 80 to 70 from baseline to 6 months, a 2.2-point increase in MCS would be expected.

**Table 3:** Models for SF-12 MCS and PANSS total score and insight level

Parameter	Estimate	SE	***P*** value
Model with baseline data			
Intercept	35.86	0.54	< 0.0001
PANSS total score			
When score < 90	−0.26	0.02	< 0.0001
When score ≥ 90	−0.02	0.06	0.6958
PANSS Item G12 ≥ 5	8.63	1.01	< 0.0001
Model with longitudinal data			
Intercept	35.23	2.18	< 0.0001
Visit 12: 12 months vs 6 months	0.24	0.47	0.6142
Baseline MCS	0.44	0.03	< 0.0001
Baseline PANSS total score	−0.15	0.02	< 0.0001
Change in PANSS total score from baseline	−0.22	0.02	< 0.0001
Follow-up PANSS Item G12 ≥ 5	4.48	1.23	0.0003

*Abbreviations*: *MCS* Mental Component Summary, *PANSS* Positive and Negative Syndrome Scale, *SE* standard error

### Relationship between patient-reported SF-12 MCS score change and insight

The association between level of insight impairment and SF-12 MCS score was examined. Patients with poor insight (PANSS Item G12 rating ≥ 5) reported an 8.63-point higher SF-12 MCS score (*P* < 0.0001) at baseline and a 4.48-point higher score (*P* = 0.0003) at follow-up compared with patients with good or fair insight (PANSS Item G12 rating < 5; Table 3).

## Discussion

This post hoc analysis of CATIE study data provides a greater understanding of how the degree of insight may influence responses of individuals with schizophrenia on patient-reported outcomes, including health-related QoL measures. At baseline, statistically significant associations were observed between CATIE participants’ levels of insight, based on PANSS Item G12 rating, and most outcomes assessed, including patient-reported health-related QoL outcomes, illness severity, PANSS component scores, and depression scale scores. Among CATIE participants, insight that was moderately severe or worse (i.e., PANSS Item G12 rating ≥ 5) was associated with better mental/emotional health status and better health-related QoL (based on SF-12 MCS and PCS scores) compared with those with less impairment (PANSS Item G12 rating < 5). Patients with more impaired insight (i.e., PANSS Item G12 ≥ 5) accounted for 10% of CATIE participants and had lower levels of baseline depression compared with patients with less impaired insight (i.e., PANSS Item G12 rating < 5). Also, among the group with poor insight, there was a discrepancy in appraisal of schizophrenia severity, with lower severity reported by patients than by physicians. These results are consistent with the finding that, compared with the overall CATIE population, patients with substantially greater insight impairment were less likely to adhere to their medication [39]. For the 90% of CATIE patients with baseline PANSS Item G12 rating < 5, physician- and patient-reported severity ratings were generally more consistently close in their agreement with one another.

The validity of patient-reported outcomes to predict health outcomes in schizophrenia has been questioned because of possible confounding due to impaired insight, such that patients with poor insight might lack the ability to appraise their own health-related QoL accurately [40, 41]. Some studies have shown that poor insight has a weaker correlation with self- and reviewer-rated health-related QoL measurements than good insight [42, 43]. However, other studies have found that self-reported health-related QoL measures in patients with schizophrenia are reliable [44], although this appears to be most reproducible among those with better insight (and potentially less severe symptoms) [45]. The current results indicate that although severe insight impairment can be associated with significant divergence between physician- and patient-reported ratings of illness, the proportion of patients in which this occurs is likely small. For a large majority of patients with schizophrenia—90% in the current analysis—physician and patient ratings aligned. These findings have important implications for the use of patient-reported outcomes in the clinical trials enrolling patients with schizophrenia. Because successful treatment of schizophrenia requires functional recovery in addition to remission of symptoms [46], assessments of health-related QoL and daily functioning are essential for evaluating treatment efficacy in patients with schizophrenia [47]. Impairment in insight would not be expected to have a substantial effect on measurement of those outcomes at a study population level, as patient-reported outcomes diverge from physician-reported outcomes only with moderate-severe to extreme impairment in insight. However, on the individual patient level, patients with very poor insight may report better mental health status relative to those with fair or good insight, and this should be taken into consideration when interpreting such data.

In the current post hoc analysis, self-reported mental health (SF-12 MCS) status was found to be linearly related to symptom severity (PANSS total score) when PANSS was <90, but no relation was found in patients with greater symptom severity (≥90). Linear regression modeling demonstrated a negative association between self-reported mental health and PANSS total scores, as expected given that higher SF-12 MCS scores indicate better health-related QoL, whereas higher PANSS scores indicate greater symptom severity. For both baseline and longitudinal data, an approximate 2-point change in SF-12 MCS was associated with a 10-point change in PANSS total score. No relationship was observed between self-reported physical health (SF-12 PCS score) and PANSS total score.

In this analysis, we found that depression scores (based on Calgary total score) decreased with increasing impairment in insight. The change across the insight continuum was small but consistent with the findings from a recent meta-analysis, in which a significant association between global clinical insight and depression was observed, with better insight associated with higher levels of depressive symptoms [48]. While the current post hoc analysis (in line with other studies [3, 13, 31]) reports better health-related QoL among patients with schizophrenia with impaired insight relative to those with fair or good insight, other studies have found that patients with impaired insight have decreased health-related QoL [42, 49] or have found no significant association between the two [28, 50–52]. Several factors may explain the discrepancies between these findings, including differences in assessments used to measure insight [28, 31, 52]. In addition, characteristics of the study population, such as treatment history, symptom stability, status of social and living situations, and objective QoL [42, 49] may also be operant. The single measure in this analysis that did not vary significantly with insight was the Neurocognitive Composite Score; however, an association was observed between insight and the PANSS cognitive component score. Significant associations between measures of insight and cognition have been reported in meta-analyses of patients with schizophrenia (11 studies) and in patients with schizophrenia or psychosis (35 studies) [53]. However, the authors observed small and inconsistent correlations in a number of included studies and posited that the relationship between insight and cognitive deficits is nonlinear [53]. Results of published studies suggest that the relation between insight and cognition may vary with age, severity of disease, or number of previous episodes [54, 55].

Treatment of poor insight in schizophrenia has been approached using both pharmacological and psychological therapies. Clozapine and second-generation antipsychotics have been associated with improvements in insight in schizophrenia [20, 56]. However, the association was not observed in an analysis that controlled for other clinical factors, indicating that effects on impaired insight were mediated by overall improvement in symptoms [20]. Several psychological therapies have been assessed for effect on insight in schizophrenia, including cognitive behavioral therapy, psycho-education, adherence therapy, social skills training, and metacognitive training [8, 57]. Overall, meta-analyses have shown small to moderate effect sizes for these interventions on insight, although the approaches vary in effectiveness [8, 57]. Cognitive behavioral therapy for psychosis, which is specifically adapted for individuals with psychosis, has been associated with improvements in insight in several studies [58–61] and is recommended in the American Psychiatric Association practice guideline for the treatment of patients with schizophrenia [62]. Metacognitive training for psychotic illnesses and metacognitive reflection and insight therapy (MERIT) are emerging therapies that address insight in schizophrenia and aim to help patients strengthen their understanding of their distorted mental processes (through metacognitive training) or their self-appraisal abilities (through MERIT) [63–65]. One study assessing MERIT found that patients with first-episode psychosis and poor clinical insight who received 6 months of MERIT had statistically significant improvements in objective measures of insight without any increases in hopelessness or emotional distress relative to those who had standard meetings with therapists [66]. A systematic review in patients with schizophrenia spectrum disorders concluded that metacognitive training improved cognitive insight, illness awareness, and awareness of delusions and hallucinations, while MERIT was found to be less effective [67]. These types of therapy hold promise for patients with poor insight.

Limitations of the current study include the post hoc nature of the analysis and the use of a single-item measurement for insight, PANSS Item G12. In addition, while the goal of the current analysis was to better understand how impairment in insight might affect responses on patient-reported measures of symptom severity and mental health, observed associations between the outcomes assessed do not necessarily indicate a causal relationship. Finally, the results observed here may not generalize to patients with schizophrenia who differ from those who enrolled in the CATIE study. CATIE study participants were predominantly male and white and notably were willing to enroll in a clinical trial for the treatment of schizophrenia; such patients may differ in their level of insight from those who are unwilling to enroll in a clinical study. Our analysis is strengthened by use of a nonparametric regression method (LOESS) to examine the relationship between insight and subjective health-related QoL. By characterizing relatively large groups for each of the levels of insight, this approach revealed a spectrum of insight levels based on PANSS Item G12 ratings.

In conclusion, this post hoc analysis assessed the relationship between degrees of impairment in insight and physician- and patient-reported outcomes. Results indicate that the inverse relationships between insight and self-perceived mental health and mood are most robust once moderate-severe levels of impairment of insight are reached. In this analysis, agreement about health status, depression, and neurocognition differed between patients with different degrees of insight impairment, but most notably between the 10% of patients with moderate-severe to extreme lack of insight and the 90% with lower levels of impairment. For most patients with schizophrenia enrolled in the CATIE study, there was little effect of insight impairment on the convergence of ratings for patient- versus physician-reported outcomes. By providing a greater understanding of the relationship between insight impairment and patient-reported outcomes in schizophrenia, the results from this analysis may be informative for clinicians seeking to address impairment in insight to help patients achieve their therapeutic goals.

## Data Availability

The data used in the preparation of this manuscript are proprietary to Alkermes, Inc. Alkermes, Inc. is committed to public sharing of data in accordance with applicable regulations and laws, and requests can be submitted to the corresponding author.

## Abbreviations

CATIE: Clinical Antipsychotic Trials of Intervention Effectiveness
CGI-S: Clinical Global Impression−Severity
LOESS: Locally weighted scatterplot smoothing
MCCB: MATRICS Consensus Cognitive Battery
MCS: Mental Component Summary
PANSS: Positive and Negative Syndrome Scale
PCS: Physical Component Summary
PGI-S: Patient Global Impression−Severity
QoL: Quality of life
SD: Standard deviation
SE: Standard error
SF-12: 12-Item Short-Form Health Survey
